# Triacontanol Promotes the Fruit Development and Retards Fruit Senescence in Strawberry: A Transcriptome Analysis

**DOI:** 10.3390/plants9040488

**Published:** 2020-04-10

**Authors:** Qianqian Pang, Xueqin Chen, Jinhua Lv, Teng Li, Jinggui Fang, Haifeng Jia

**Affiliations:** Key Laboratory of Genetics and Fruit Development, Horticultural College, Nanjing Agricultural University, Nanjing 210095, China; 2019104017@njau.edu.cn (Q.P.); 2019104011@njau.edu.cn (X.C.); 2019104012@njau.edu.cn (J.L.); 2019804156@njau.edu.cn (T.L.); fanggg@njau.edu.cn (J.F.)

**Keywords:** strawberry, triacontanol, development, senescence, transcriptome

## Abstract

Triacontanol (TA) is a non-toxic, pollution-free, low-cost, high-efficiency, broad-spectrum plant growth regulator that plays an important role in plant growth and development, but its regulation mechanism of strawberry (Sweet charlie, *Fragaria* × *ananassa* Duch.) fruit development is still unclear. In this study, we showed that TA treatment (50 μM) could promote fruit development by up-regulating factors related to fruit ripening-related growth and development. TA increased fruit sugar content and anthocyanin accumulation, and many stress-related enzyme activities. In the meantime, Illumina RNA-Seq technology was used to evaluate the effect of TA treatment on strawberry fruit senescence. The results showed that 9338 differentially expressed genes (DEGs) were obtained, including 4520 up-regulated DEGs and 4818 down-regulated DEGs. We performed gene ontology (GO) enrichment and Kyoto Encyclopedia of Genes and Genomes (KEGG) analysis of these DEGs. The results showed that TA treatment caused changes in transcript levels related to cellular processes, hormones and secondary metabolism, such as DNA metabolic processes, flavonoid synthesis, and plant hormone signal transduction. Bioinformatics analysis showed that many transcription factors were related to fruit maturity. Taken together, this study will provide new insights into the mechanism of strawberry development and postharvest response to TA treatment.

## 1. Introduction

Strawberry (*Fragaria* × *ananassa* Duch.) belongs to the perennial herb of the Rosaceae family. Its berries are bright in color, soft and juicy, sweet and sour, and rich in aroma, protein, organic acids and essential minerals and other nutrients. Known as the “Queen of Fruits”, it is an edible plant with both horticultural and ornamental elements, and is well received by consumers [1]. Strawberry is a typical non-climacteric fruit. It has a high water content, but is fragile and susceptible to environmental factors. It is susceptible to aging and rot postharvest, and has a short storage period [2,3]. Fruit maturation and aging is a complex and orderly biochemical process. Not only the color, but the texture, aroma, flavor and other metabolisms also will change postharvest. Tissue appearance traits are also highly coordinated and controlled by their inherent genes [4,5,6,7].

Triacontanol (TA) is a long-chain fatty alcohol composed of 30 carbon atoms. The chemical name is triacontanol-1 or n-triacontanol, referred to as triacontanol or TA or TRIA, also known as benzyl alcohol. Its chemical formula is: CH_3_ (CH_2_)_28_CH_2_OH. In 1933, Chibnall and others first isolated TA from alfalfa and regarded it as the main component of alfalfa leaf wax. Afterwards, TA was extracted from many plant waxes and insect waxes, and artificially synthesized finally. But its effect on plants was not known, but just a chemical agent [8]. In 1975, Ries discovered that TA has certain physiological activities. After a series of experiments, it was found that TA has an effect of increasing yields of corn, rice, wheat, tomato, carrot, cucumber, lettuce, soybean, etc. In 1977, Ries et al. announced that TA was a new naturally plant growth regulator [9]. Ries’ discovery has aroused great interest and attention of other scholars in many countries. Studies have shown that TA is a broad-spectrum plant growth regulator [10]. TA can increase the activities of nitrate reductase, amylase, peroxide isoenzyme [11], promote carbon-nitrogen metabolism, and increase the carbon-nitrogen ratio and the storage and accumulation of ATP to increase production [12]. TA can promote the absorption of water and mineral elements by plants, as well as significantly increase the content of chlorophyll and the net photosynthetic rate of leaves, promote the flow of more photosynthetic products to crop seeds and fruits, and accelerate the transport of organic substance stored in the stem to the grains. It can regulate the transport, distribution and accumulation of photosynthetic products [13,14]. Many studies have shown that TA can also regulate the plant’s resistance to abiotic stress, such as water stress, salt stress, drought and high temperature stress [15,16,17,18]. In addition, TA can delay the senescence of rice leaves, cut tulips, and improve freshness. Its long carbon chain structure acts easily on the cell membrane, changes the composition and structure of the cell membrane, promotes material movement, accelerates plant growth, and delays aging [19,20,21]. TA has been extensively and intensively studied in regulating plant growth and development and improving stress resistance, but there are few reports on its regulation of fruit senescence.

In order to know the effect of TA on strawberry fruit development and postharvest storage, we analyzed the physiological index changes after TA treatment on the strawberry plant, and molecular changes after TA treatment on the initial ripening (IR, 16 days after flowering) strawberry fruit senescence using RNA-sequencing. We performed transcriptome sequencing on octaploid strawberries and obtained high-quality data. In total, we identified a number of differentially expressed genes (DEGs), performed gene ontology (GO) and Kyoto Encyclopedia of Genes and Genomes (KEGG) functional annotations, and analyzed biological pathways affected by TA. The genetic information and molecular mechanism provided by this study will help to understand the effect of TA on strawberry senescence, and have guiding significance for strawberry storage and preservation.

## 2. Results

### 2.1. Effects of TA on Strawberry Fruit Development

**Figure 1:**
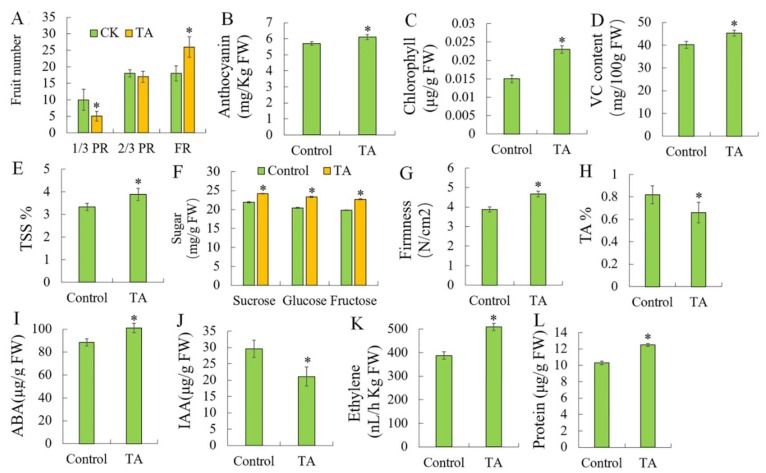
Effects of triacontanol on strawberry fruit ripening. (**A**). Fruit number. (**B**). Anthocyanin content. (**C**). Chlorophyll content. (**D**). VC (vitamin C) content. (**E**). TSS % (total soluble solid). (**F**). Sugar content. (**G**). Firmness. (**H**). TA %. (**I**). ABA (abscisic acid) content. (**J**). IAA (indoleacetic acid) content. (**K**). Ethylene content. (**L**). Protein content. Control: CK; TA: triacontanol. Vertical bars represented standard deviations (SD) of means (*n* = 3). Asterisks indicated statistically significant differences at *p* < 0.05 as determined by Student’s *t*-test.

To determine the role of TA in strawberry fruit development, we chose 20 strawberry plants for treatment with TA and the fruit development was observed. Sterile water was used as a control (CK). Twenty-eight days after treatment, we observed the ratio of full red, 2/3 red, and 1/3 red fruits. TA and water-treated fruits showed ratios of 18:18:10, 26:17:5, respectively (Figure 1A). Thus, exogenous TA promoted coloration compared to the control, which was supported by anthocyanin contents (Figure 1B). However, TA also increased the chlorophyll content (Figure 1C), vitamin C content (Figure 1D), and total soluble solid compared to the control fruit (Figure 1E). TA led the fruit to accumulate sugars such as sucrose, glucose and fructose (Figure 1F), and decreased the total acid content. These changes all indicated that TA promoted the strawberry fruit ripening process. To know the effects of TA on fruit coloration, the hormone contents that were related to fruit ripening, including ethylene (Figure 1K), abscisic acid (ABA) (Figure 1I), and indoleacetic acid (IAA) (Figure 1J), were determined. The results showed that exogenous TA increased ethylene emission and ABA accumulation and decreased IAA accumulation compared to the control. The protein content also increased by TA (Figure 1L). These changes also affected the fruit firmness; TA delayed the fruit softening (Figure 1G). In total, these indicated that the effects of TA on the coloration of strawberry fruit might be associated with ABA, ethylene, and IAA that promoted the fruit ripening process.

### 2.2. Effects of TA on Sugar Metabolism

**Figure 2:**
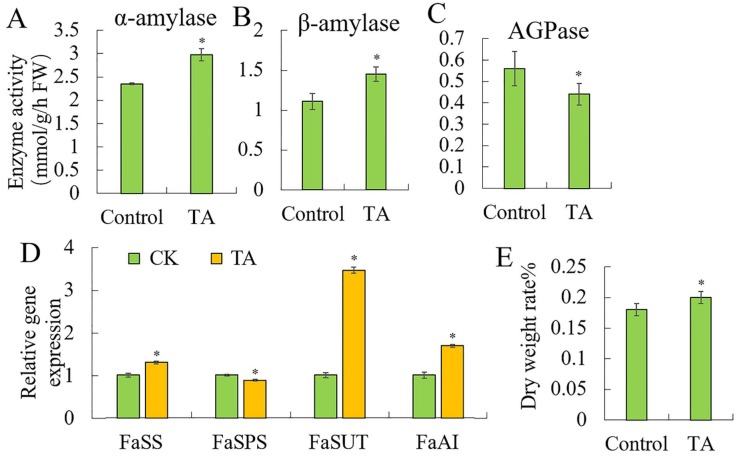
Effects of triacontanol on sugar metabolism. Enzyme activities of (**A**). α-amylase. (**B**). β-amylase. (**C**). AGPase. (**D**). Relative gene expression. (**E**). Dry weight rate%. Vertical bars represented standard deviations (SD) of means (*n* = 3). Asterisks indicated statistically significant differences at *p* < 0.05 as determined by Student’s *t*-test.

To know the effect of TA on the sugar metabolism, we measured the starch and sugar metabolism related enzymes activities and genes expression. The results showed that TA could promote starch degradation by increasing the starch degrading enzyme activities of α-amylase (Figure 2A) and β-amylase (Figure 2B), but inhibits the starch synthase enzyme activity of ADP-glucose pyrophosphorylase (AGPase) (Figure 2C). These changes led to more starches degrading into sugars. Meanwhile, the TA also increased expression of the sucrose transport gene *SUT1* (Figure 2D), which led to more photosynthetic sucrose being transported to the fruit inside. The sucrose synthase *SS* and acid invertase *AI* were increased by TA, but the sucrose phosphatase synthase *SPS* was decreased by TA, suggesting that sucrose was degraded into the glucose and fructose. These changes indicated that TA-promoted starch degraded into sugars in the fruit, which increased the fruit’s dry weight (Figure 2E) and promoted the fruit ripening process.

### 2.3. Effects of TA on Strawberry Fruit Stress Resistance

**Figure 3:**
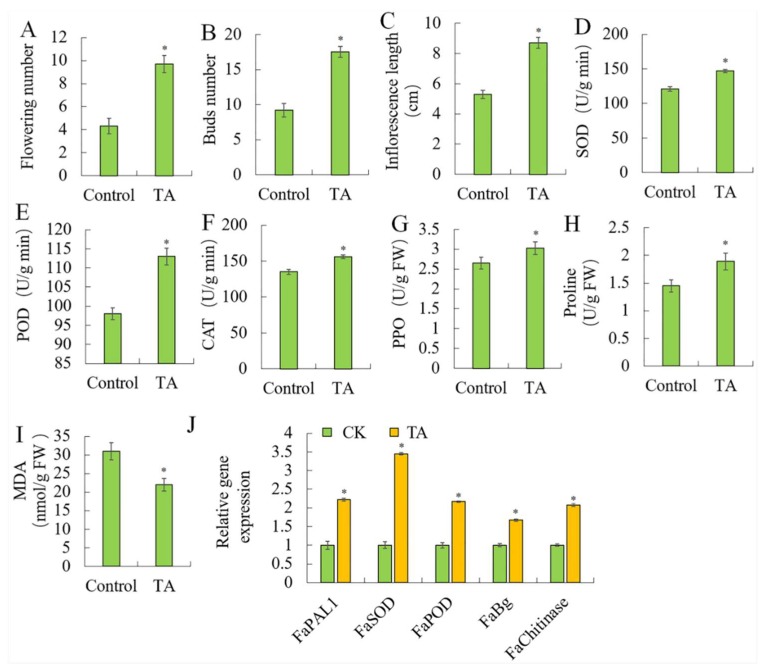
Effects of triacontanol on strawberry fruit stress resistance. (**A**). Number of flowers. (**B**). Number of buds. (**C**). Inflorescence length. Enzyme activities of (**D**). SOD (superoxide dismutase). (**E**). POD (peroxidase). (**F**). CAT (catalase). (**G**). PPO (polyphenol oxidase). (**H**). Proline. (**I**). MDA (malondialdehyde). (**J**). Relative gene expression. Vertical bars represented standard deviations (SD) of means (*n* = 3). Asterisks indicated statistically significant differences at *p* < 0.05 as determined by Student’s *t*-test.

TA also affected the fruit development. TA increased the number of flowers per fruit (Figure 3A), such that the water-treated plants had an average of 4.3 flowers per plant, and the TA-treated plants had 9.7, and the number of buds (Figure 3B) and the inflorescence length (Figure 3C) were also increased. TA protected the fruit from external damage through increased enzyme activities, such as superoxide dismutase SOD (Figure 3D), peroxidase POD (Figure 3E), catalase CAT (Figure 3F) and polyphenol oxidase PPO (Figure 3G). The stress-related genes, *FaPAL*, *FaSOD*, *FaPOD*, *FaBg* and *FaChitinase* were also increased by TA (Figure 3J). Meanwhile, TA increased the proline content (Figure 3H), but decreased the malondialdehyde MDA content (Figure 3I). These suggested that TA plays an important role in maintaining the integrity of membrane structure, eliminating free radicals and alleviating membrane lipid peroxidation injuries. It can alleviate and improve the adaptability of plants to adversity.

### 2.4. RNA Sequencing Analysis

In order to obtain the required transcriptome information, after extracting, purifying, and constructing a library of RNA from the fruits of TA-treated and control strawberry fruits, these libraries were paired-end (PE) and sequenced using Next-Generation Sequencing (NGS) based on the Illumina HiSeq sequencing platform. TA-treated samples and control samples produced an average of 45.93 million raw reads and 46.08 million raw reads, respectively (Supplementary Materials Table S1). The Q30 (%) of the two groups of samples (the percentage of bases with a base recognition accuracy rate above 99.9%) was not less than 94.9%. After removing some low-quality reads with joints, high-quality sequence reads (clean reads) were obtained. The TA-treated group and the control group averaged approximately 42.72 million and 42.83 million clean reads, respectively. The percentage of clean reads in sequencing reads in both groups was not less than 92.62%. The data sets of the TA-treated group and the control group were not less than 38.95 million, and the tag density was sufficient for the quantitative analysis of gene expression.

The upgraded HISAT software of TopHat2 was used to compare the filtered reads to the reference genome. The percentage of the reference genome sequence (Total_Mapped) and the sequence aligned to only one position (Uniquely_Mapped) in the TA-treated group were 91.73%, 91.75%, 91.90% and 86.87%, 86.86%, 86.82%, respectively (Supplementary Materials Table S1). In the control group, the percentage of the sequence of the reference genome (Total_Mapped) and the percentage of sequences aligned to one position (Uniquely_Mapped) were 90.91%, 91.16%, 91.20%, and 86.68%, 86.76%, and 86.78%, respectively. The percentage of the total number of sequences in the TA-treated group and the control group that were only aligned to one position was not less than 86.68%. The distribution of reads compared to the genome was calculated, the percentages of reads compared to the gene region in the TA-treated group and the control group were 96.06%, 96.16%, 96.22% and 95.38%, 95.73%, 95.66%, respectively.

#### 2.4.1. Expression Analysis

**Figure 4:**
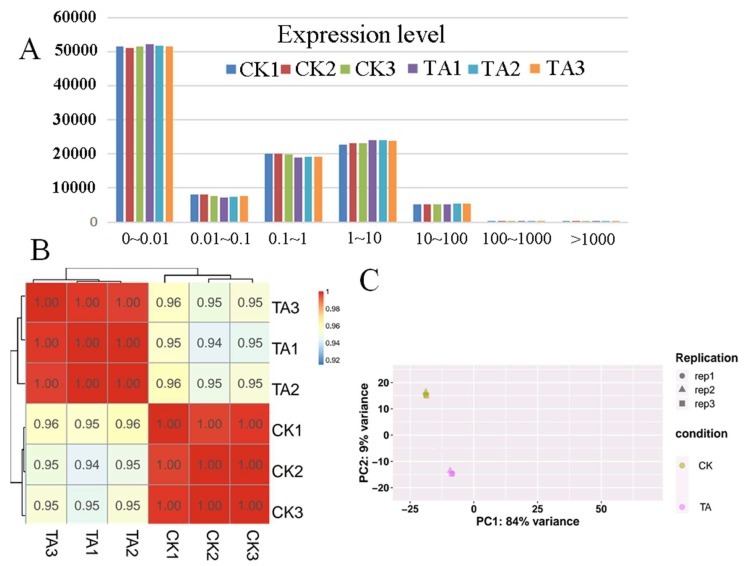
Different treatment samples analysis. (**A**). The calculation of expression level in strawberry fruit under TA (triacontanol) treatment. A total of 108,087 genes were identified and the number of genes in different sections were similar among both control and TA-treated. (**B**). Correlation matrix showed the correlation between samples. Both the CK (control) and TA-treated had the darkest colors, indicating that the correlation between the same treatment samples was the highest. (**C**). Principal Component Analysis (PCA) were performed on the biological replicates of each sample set (CK and TA), which the same treatment samples were closed, indicating the higher similarity between samples.

We used HTSeq statistics to compare the Read Count value of each gene as the original expression of the gene. In order to make the gene expression levels of different genes and samples comparable, we used Fragments Per Kilo bases per Million fragments (FPKM) to standardize the expression level. In the reference transcriptome, we generally thought that genes with FPKM > 1 were expressed. An average of 29,695 genes were expressed in the TA-treated group and 28,769 in the control group, respectively. According to the expression calculation table, the expression was divided into different intervals, and the number of genes in different expression intervals of each sample was counted. The number of genes in the same expression interval of the control group and the TA-treated group was similar (Figure 4A, Supplementary Materials Table S2). In order to ensure the rigor of the experiment, we calculated the Pearson correlation coefficients of all gene expression levels between each sample, and reflected these coefficients in the form of a correlation matrix heat map (Figure 4B). At the same time, each sample was compared using principal component analysis (PCA) (Figure 4C). The results showed that the sampling of this experiment was reasonable and suitable for the next analysis.

#### 2.4.2. Differential Expression Genes Analysis

**Figure 5:**
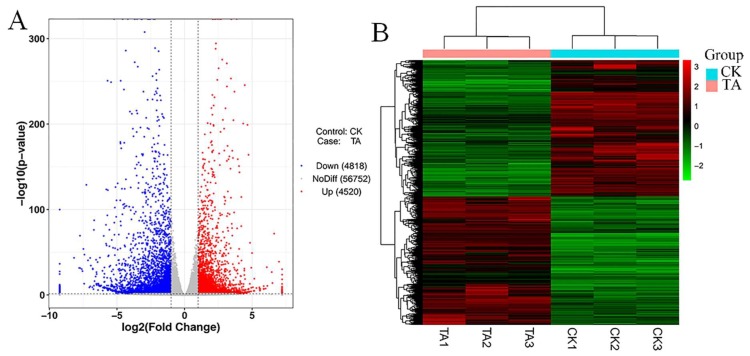
Classification analysis of differentially expressed genes. (**A**). Volcano map displayed the relation between log 2 (Fold Change) and -log 10 (*p*-value), which more obviously showed the diversity expression of up-regulated and down-regulated DEGs (differentially expressed genes). The blue color on the figure were down-regulated genes, the red were up-regulated genes and the grey color in the middle were the genes have no difference. (**B**). Clusters of differentially expressed genes in the control and TA-treated groups. Genes were shown horizontally, and each column was a sample. The red was highly expressed genes and green was low expressed genes.

DESeq was used for differential analysis of gene expression, and the conditions for screening differentially expressed genes were: multiple of expression difference | log2FoldChange | > 1 and significance *p*-value < 0.05. Among all the detected 66,090 genes (Supplementary Materials Table S3), the expression levels of 56,752 genes were not significantly changed, and 9338 genes were differentially expressed under the action of TA, of which 4520 genes (48.40% of 9338) were up-regulated and 4,818 genes (51.60% of 9338) were down-regulated, respectively. The volcanic map of the differentially expressed genes was drawn using the R ggplots2 software package to further analyze the distribution of DEGs (Figure 5A), showing the fold difference of gene expression and significance of results. From the figure we got that the distribution of up-regulated and down-regulated genes was roughly symmetrical. We also used the R language Pheatmap package to perform a two-way cluster analysis on the union and samples of the differential genes of all the comparison groups (Figure 5B). The expression pattern of differentially expressed genes was under different experimental conditions to explore unknown biological connections between genes.

#### 2.4.3. GO and KEGG Analysis

The gene ontology (GO) database was used for enrichment analysis to find the GO term in which the differential genes were significantly enriched compared to the entire genome background, so as to determine the main biological function of the differential genes. Gene ontology was divided into three major functional categories: molecular function (MF), cellular component (CC), and biological process (BP). This GO enrichment analysis had annotated 3705 Term (Supplementary Materials Table S4), of which 2262 belong to BP (61% of 3705), MF has 1092 (29.5% of 3705), and CC has 351 (9.5% of 3705). In the functional category of BP, biological process (GO: 0008150) and metabolic process (GO: 0008152) had the highest number of differential genes, which were 3334 and 2473, respectively; the term with the highest number of differential genes in CC category was cellular component (GO: 0005575,2671 DEGs) and membrane (GO: 0016020,1593 DEGs); in the MF category, molecular function (GO: 0003674) and catalytic activity (GO: 0003824) had the highest number of differential genes, that were 3600 and 2372, respectively.

**Figure 6:**
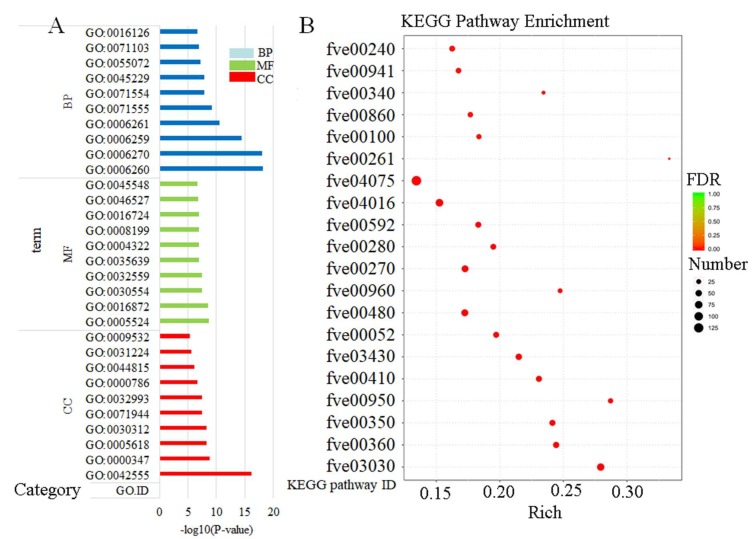
Enrichment analysis of differentially expressed genes. (**A**). The top ten significant enrichment GO term in biological process (BP), molecular function (MF) and cellular component (CC). The abscissa was the logarithm of the significant enrichment *P* value with the base 10, and the ordinate was the gene ID. (**B**). Top 20 KEGG enrichment pathways by FDR. Rich Factor was the ratio of the differentially expressed number of genes located in the pathway. The higher the Rich Factor, the higher the degree of enrichment. FDR was false discovery rate in the range of 0 to 1, the closer to zero, the more significant the enrichment.

DEGs were significantly enriched (*p* ≤ 0.05) to 674 GO terms. The main terms of BP (372) were DNA replication (GO: 0006260), DNA replication initiation (GO: 0006270), and DNA metabolic process (GO: 0006259). The main terms of the cell component CC (63) were MCM complex (GO: 0042555), THO complex (GO: 0000347), cell wall (GO: 0005618). The main terms of molecular function MF (239) were ATP binding (GO: 0005524), intramolecular lyase activity (GO: 0016872), adenyl nucleotide binding (GO: 0030554). The top 10 most significant GO term entries in each GO category were selected for display (Figure 6A).

In addition, in order to analyze the biological pathways of DEGS, DEGS was enriched into 124 KEGG pathways using the Kyoto Encyclopedia of Genes and Genomes (KEGG) annotations (Supplementary Materials Table S5). These KEGG pathways fell into six categories: Metabolism (73.3%), Genetic Information Processing (16.9%), Environmental Information Processing (3.2%), Cellular Processes (3.2%), Organical Systems (1.6%), and Human Diseases (1.6%). In order to evaluate the functional role of TA response genes in different biochemical pathways, we determined the impact pathways of TA application based on expression profiling. After TA treatment, 38 biochemical pathways were significantly affected (*p* value < 0.05). These pathways included the biosynthesis or degradation of various metabolites, including sugars and polysaccharides, lipids such as amino acids, fatty acids, cofactors and vitamins, and some secondary metabolites.

Among all KEGG pathways, the first six pathways containing the most DEGs were “Plant hormone signal transduction” (fve04075), “Phenylpropanoid biosynthesis” (fve00940), and “Plant-pathogen interaction” (fve04626), “Protein processing in endoplasmic reticulum” (fve04141), “MAPK signaling pathway-plant” and “Starch and sucrose metabolism” (fve00500). The first six pathways with the most significant enrichment (with the lowest FDR value) were DNA replication (fve03030), Phylnalanine metabolism (fve00360), Tyrosine metabolism (fve00350), Isoquinoline alkaloid biosynthesis (fve00950), Mismatch repair (fve03430), and beta-Alanine metabolism (fve00410). The top 20 KEGG pathways with the most significant enrichment were now shown (Figure 6B).

#### 2.4.4. Effects of TA-Treated on Antioxidant Enzymes in Fruits

The accumulation of reactive oxygen species (ROS) is an important factor to promote strawberry fruit senescence [22,23]. TA could regulate the activity of antioxidant enzymes such as superoxide dismutase (SOD), catalase (CAT), peroxidase (POD), and polyphenol oxidase (PPO), and then affect the fruit antioxidant capacity (Table 1, Supplementary Materials Table S3). Transcriptome data showed that SOD activity was enhanced by five up-regulated genes after TA treatment, one of which was Cu/Zn-SOD and four genes were Fe-SOD. At the same time, positive regulation of superoxide dismutase activity was also induced by three up-regulated genes. One up-regulated gene enhanced the activity of CAT. 14 up-regulated genes, eight down-regulated genes worked together on POD, one up-regulated gene and five down-regulated genes jointly regulated PPO activity, POD activity increased, and PPO activity decreased. TA treatment enhanced the activities of SOD, CAT, and POD, decreased the activity of PPO, and improved strawberry’s resistance to ROS.

#### 2.4.5. Effect of TA-Treated on Softening

Fruit firmness and crispness are all related to fruit softening. Softening is closely related to changes in the structure and composition of fruit cell walls and related enzyme activities [24]. According to transcriptome data, we identified the following softening-related enzymes (Table 2, Supplementary Materials Table S4): cellulase (Cx), alpha-L-arabinofuranosidase (α-Af), pectinesterase (PE), polygalacturonase (PG), pectate lyase (PL), beta-galactosidase (β-Gal), expansin (EXP), and xyloglucosyl transferase (XET), etc. In the GO annotation, cellulase activity was co-regulated by one up-regulated gene and six down-regulated genes, three down-regulated genes inhibited α-Af activity, nine up-regulated genes and 23 down-regulated genes worked together on PE, and PG was affected by 10 up-regulated genes and five down-regulated genes, the number of up-regulated and down-regulated genes that control PL was three, and the number of β-Gal up-regulated and down-regulated genes was seven and six, respectively. After TA treatment, eight genes of EXP were up-regulated, 13 genes were down-regulated, 16 genes of XET were up-regulated, and three genes were down-regulated. The results showed that TA inhibited the activities of Cx, α-Af, PE, and EXP, and promoted the activities of PG, β-Gal, and XET, and the effects on PL activities needed further exploration. More down-regulated genes suggesting that TA may delay the softening process of strawberry fruits.

#### 2.4.6. Effect of TA on Pigment Metabolism

The accumulation of pigment is the material basis for the formation of peel color. The type and content of pigment ultimately determine the color of the peel. Peel pigments are mainly composed of chlorophyll, carotenoids, flavonoids, and anthocyanins, and there may be one pigment or multiple pigments in the same fruit [25].

Chlorophyll is the main pigment for photosynthesis in plants. In KEGG analysis of porphyrin and chlorophyll metabolism pathway, 32 DEGs were significantly enriched, of which 28 genes were up-regulated and 4 genes were down-regulated (Table 3, Supplementary Materials Table S5). A series of enzyme activities enhanced in chlorophyll biosynthesis, such as encoding magnesium chelatase subunit (chlD, chlI), protoporphyrinogen/coproporphyrinogen III oxidase (PPOX), magnesium-protoporphyrin O-methyltransferase (chlM), vitamin chlorophyllide a 8-vinyl-reductase (DVR), chlorophyll/bacteriochlorophyll a synthase (chlG), chlorophyllide a oxygenase (CAO) and other genes were all up-regulated. During chlorophyll degradation, one gene of 7-hydroxymethyl chlorophyll a reductase (HCAR) was up-regulated and two genes encoding chlorophyll (ide) b reductase (NOL, NYC1) were up-regulated. TA treatment promoted the biosynthesis and degradation of chlorophyll. From the above enzymes with active changes and the specific data in Table 3, the effect on synthesis may be greater than the effect on degradation.

In the carotenoid biosynthetic pathway, 23 DEGs were significantly enriched, of which 9 genes were up-regulated and 14 genes were down-regulated (Table 4, Supplementary Materials Table S5). The carotenoid rate-limiting enzyme phytoene synthase (PSY) was reduced by two down-regulated genes. Both violaxanthin de-epoxidase (VDE) and capsanthin/capsorubin synthase (CCS1) were controlled by one down-regulated gene. Seven down-regulated genes acted on 9-cis-epoxycarotenoid dioxygenase (NCED); both of prolycopene isomerasecrt (crtISO) and abscisic-aldehyde oxidase (AAO3) had two up-regulated genes, beta-carotene 3-hydroxylase (CrtR-b) and abscisate beta-glucosyltransferase (AOG) were also differentially expressed, and both of them had 1 up-regulated gene. (+)-abscisic acid 8’-hydroxylase (CYP707A) activity was jointly regulated by 3 up-regulated genes and 3 down-regulated genes. TA treatment inhibited PSY, VDE, CCS1, and NCED activities, and promoted crtISO, AAO3, CrtR-b, and AOG activities. More genes including the rate-limiting enzyme PSY were down-regulated, we believe that TA treatment may inhibit carotenoid biosynthesis.

Anthocyanins are a class of flavonoids. The color of strawberry fruits is mainly regulated by anthocyanins content [26]. In the phenylpropane metabolic pathway, 8 genes encoding phenylalanine ammonia-lyase 1 (PAL1) were down-regulated (Table 5). In Flavonoid biosynthesis, 35 DEGs were significantly enriched, of which 1 gene was up-regulated and 34 genes were down-regulated (Supplementary Materials Table S5). One gene encoding caffeoyl-CoA O-methyltransferase (CCoAOMT) was up-regulated, genes encoding trans-cinnamate 4-monooxygenase (CYP73A, 4 down-regulation), chalcone synthase (CHS, 7 down-regulation), shikimate O-hydroxycinnamoyl transferase (HCT, 5 Down-regulation), 5-O-(4-coumaroyl)-D-quinate 3’-monooxygenase (C3’H, 1 down-regulation), chalcone isomerase (CHI, 3 down-regulation), bifunctional dihydroflavonol 4-reductase/flavanone 4-reductase (DFR, 4 down-regulation), naringenin 3-dioxygenase (F3H, 3 down-regulation), leucoanthocyanidin reductase (LAR, 4 down-regulation), anthocyanidin synthase (ANS, 3 down-regulation) were down-regulated. The genes encoding UDP-glucose flavonoid 3-O-glucosyltransferase (UFGT) was also differentially expressed, with 5 genes up-regulated and 6 genes down-regulated. TA treatment promoted CCoAOMT activity and inhibited CYP73A, CHS, HCT, C3’H, CHI, DFR, F3H, LAR, ANS, and UFGT activities. Apparently TA inhibits anthocyanin synthesis.

Triacontanol retards the change of strawberry fruit color by promoting chlorophyll synthesis and inhibiting carotenoid and anthocyanin synthesis.

#### 2.4.7. Effects of TA Treatmen on Hormones

Whether before or after harvest, fruit maturation is affected by plant hormones [27]. In the plant hormone signal transduction pathway, 133 DEGs were significantly enriched, of which 61 genes were up-regulated and 72 genes were down-regulated (Table 6, Supplementary Materials Table S5). Based on transcriptome data, many genes were involved in auxin, cytokinin (CTK), gibberellin (GA), abscisic acid (ABA), ethylene (ETH), brassinolide (BR), jasmonate (JA) and salicylic acid (SA) signal transduction. In auxin signal transduction, the down regulated genes inhibited the activities of auxin influx carrier (AUX1) (1 down-regulated), transport inhibitor response 1 (TIR1) (2 down-regulated) and auxin response factor (ARF) (1 down-regulated). The activities of auxin responsive protein IAA (Aux/IAA) (9 up-regulated, 4 down-regulated), auxin responsive GH3 gene family (GH3) (6 up-regulated, 3 down-regulated) and Saur family protein (Saur) (8 up-regulated, 5 down-regulated) were found. After TA treatment, most of the positive genes including aux responsive protein IAA (Aux/IAA) were up-regulated, while transport inhibitor response 1 (TIR1) was down regulated. This suggesting that TA treatment may promote IAA synthesis and auxin signal transduction to promote the early expansion of strawberry fruit and delay the senescence of strawberry fruit. In CTK signal transduction, “cytokinin receptor Arabidopsis histidine kinase 2/3/4” (CRE1) (2 down-regulated) and histidine-containing phosphotransfer protein (AHP) (1 down-regulated) were down-regulated, two -component response regulator ARR-B family (ARR-B) (3 up-regulated, 1 down-regulated) and two-component response regulator ARR-A family (ARR-A) (5 up-regulated, 1 down-regulated) were found. In GA signal transduction, two genes encoding phytochrome-interacting factor 4 (PIF4) were down-regulated. Short-chain type dehydrogenase/reductase (SDR) (3 up-regulated) and abscisic-aldehyde oxidase (AAO3) (2 up-regulated), the key enzymes in ABA biosynthesis and biosynthesis, had enhanced activity; in ABA signal transduction, the activities of abscisic acid receptor PYR/PYL family (PYR/PYL) (1 up-regulated, 8 down-regulated) and ABA responsive element binding factor (ABF) (1 down-regulated) decreased. The activities of the negative regulators "protein phosphatase 2C" (PP2C) (5 up-regulated, 1 down-regulated) and serine/threonine-protein kinase SRK2 (SNRK2) (3 up-regulated, 2 down-regulated) enhanced. The results showed that although TA promoted the synthesis of abscisic acid, it inhibited its signal transduction, which may be one of the important reasons for delaying strawberry senescence by TA treatment. TA treatment also inhibited the activities of 1-aminocyclopropane-1-carboxylate synthase (ACS) (4 down-regulated) and 1-aminocyclopropane-1-carboxylic acid oxidase (ACO) (4 up-regulated, 5 down-regulated) in ethylene biosynthesis. In ETH signal transduction, the activities of ethylene receptor (ETR) (4 down-regulated), mitogen-activated protein kinase kinase (SIMKK) (2 down-regulated), ethylene-insensitive protein 3 (EIN3) (3 down-regulated), EIN3-binding F-box protein (EBF1/2) (1 down-regulated) and ethylene-responsive transcription factor 1 (ERF1) (6 down-regulated) decreased, mitogen-activated protein kinase 6 (MPK6) (2 up-regulated) and ethylene-insensitive protein 2(EIN2) (2 up-regulated) were found. This suggesting that TA treatment may delay the senescence of strawberry fruits by inhibiting ethylene biosynthesis and signal transduction. In BR signal transduction, BRI1 kinase inhibitor 1 (BKI1) (3 up-regulations) and BR-signaling kinase (BSK) (4 up-regulated and 3 down-regulated) were up-regulated, and cyclin D3 (CYCD3) (1 up-regulated, 3 down-regulated) was down-regulated. In JA signal transduction, 1 gene of jasmonic acid-amino synthetase (JAR1) was up-regulated, 1 gene of coronatine-insensitive protein 1 (COI1) was down-regulated, 4 genes of jasmonate ZIM domain-containing protein were up-regulated, and six genes were down-regulated. In SA signal transduction, 4 genes encoding transcription factor TGA (TGA) were up-regulated and 8 genes were down-regulated.

#### 2.4.8. Effect of TA on Sugar Metabolism

After treatment with TA, 76 DEGs were identified in starch and sucrose metabolism, of which 44 were up-regulated and 32 were down-regulated (Table 7, Supplementary Materials Table S5). Hexokinase (HK), sucrose synthase (SS), starch synthase (firm SSY), granule-bound starch synthase (GBSS), glycogen phosphorylase (PYG), 1, 4-alpha-glucan branching enzyme (GBE1), alpha, alpha-trehalase (TREH) and glucan. The activity of endo-1,3-beta-glucosidase 5/6 (GN5_6) was enhanced by the up-regulated genes, and the number of up-regulated genes was 1, 8, 2,3, 2, 5, 1 and 1, respectively. Beta-fructofuranosidase (INV), endoglucanase (EGs), UTP-glucose-1-phosphate uridylyltransferase (UGP2) and glucose-1-phosphate adenylyltransferase (glgC) showed decreased activity under the influence of down-regulated genes, and the number of down-regulated genes was 3, 6, 1 and 1, respectively. The activities of beta-glucosidase (bgl), glucan endo-1,3-beta-glucosidase 1/2/3 (GN1_2_3), sucrose-phosphate synthase (SPS), trehalose 6-phosphate synthase/phosphatase (TPS), trehalose 6-phosphate phosphatase (TPP) and beta-amylase (BAM) were co-regulated by up-regulated and down-regulated genes. Among them, GN1_2_3, TPP and BAM activities were enhanced, while bgl, SPS and TPS activities were decreased. The activity of the enzyme SPS in the sucrose synthesis and the enzyme INV that catalyzes the degradation of sucrose decreased, and the SS activity increased. It is generally believed that the main function of SS is to provide precursors for the synthesis of polysaccharides in the direction of sucrose degradation. Sucrose synthase plays an important role in plant starch synthesis, improving plant stress resistance, and affecting plant growth [28,29,30]. The activities of key enzymes SSY, GBSS, SS, GBE1 in starch synthesis were enhanced, and the enzyme BAM activity of starch hydrolysis was also enhanced. TA treatment promoted starch synthesis and degradation, which may have a greater effect on synthesis than degradation. The activities of EGs and bgl, which catalyze cellulose hydrolysis, were reduced, and TA also inhibited cellulose degradation. As an important carbohydrate storage during fruit development, starch is very important in maintaining the fruit’s firmness and normal growth and development [31]. TA treatment not only affected sugar metabolism in fruits, but also had a positive effect in delaying softening and enhancing stress resistance.

#### 2.4.9. Transcription Factor Analysis

Through transcriptome sequencing, 53,367 genes related to transcription factors (TF) were identified (Supplementary Materials Table S6). They belonged to 58 different transcription factor families. Most of the genes encoding transcription factors were the members of the bHLH, NAC, MYB_related, ERF, and C_2_H_2_ family.

## 3. Discussion

Fruit senescence is a complex and orderly biochemical process, which is regulated by many factors, such as hormones, antioxidant enzymes, and softening enzymes.

The accumulation of reactive oxygen species is an important factor to promote strawberry fruit senescence [22,23]. As the fruit matures and senesces, superoxide anion radical (O^2−^) is produced in the metabolic process, which causes lipid peroxidation and produces MDA accumulation in cells. It destroys the structure and function of the membrane, causes electron leakage, and increases the rate of active oxygen radicals, which further accelerates the senescence of the fruit [32]. SOD is an important cytoprotective enzyme that scavenges oxygen free radicals in plants [33]. SOD converts harmful superoxide free radicals into hydrogen peroxide. CAT and peroxidase POD decompose hydrogen peroxide into completely harmless water [34]. PPO is related to the browning of fruits and crops. Reducing the content of PPO in fruits can prevent fruit browning and maintain the freshness of fruits [35,36]. TA treatment increased the activity of SOD, CAT and POD, inhibited the activity of PPO, reduced the content of MDA, increased the resistance of the fruit to oxidative stress through synergy, and delayed the senescence of the fruit. This study also found that among the up-regulated SOD genes, one gene was Cu/Zn-SOD and four were four genes Fe-SOD. Recent studies have shown that Cu/Zn SOD may be involved in plant resistance to abiotic stresses such as drought, salinity, aluminum ions, and low temperature [37,38,39,40]. Fe-SOD is closely related to plant disease resistance [41], and PPO can also Increase plant resistance to pathogens [36]. Therefore, we think that TA may also have the effect of enhancing the resistance and resistance of plants.

Strawberry fruit is low in firmness, soft and fragile, and perishable. Fruit firmness is important for strawberry quality and postharvest physiology. The strawberry’s firmness decreases rapidly during storage. The softening of the fruit involves complex physiological and biochemical processes, the degradation of cell wall materials and the destruction of the pectin-cellulose-hemicellulose structure are the beginning of cell separation and fruit softening [42]. Cellulase can promote the degradation of cellulose and thus promote the lysis of the cell wall, and promote the ripening and subsequent softening and decay of strawberries [43,44]. A-L-arabinofuranosidase (α-Af) belongs to sugar A family of hydrolase can hydrolyze non-reducing furan arabinose residues, and pectin side chain arabinose residues have an important effect on the stability of cell wall polysaccharides or cell wall structures. α-Af activity is of great significance in fruit ripening and softening [45,46,47,48]. Polygalacturonase, pectinesterase, and beta-galactosidase are the key enzymes for pectin solubilization [49]. Expansin (EXP) also has the effect of relaxing cell walls. In our study, TA treatment inhibited the activities of Cx, α-Af, PE, and EXP, and promoted the activities of PG, β-Gal, and XET. More down-regulated genes indicated that TA may delay the softening process of strawberry fruits, thereby delaying fruit senescence.

Color change is also an important feature of aging. Chlorophyll, carotenoids and anthocyanins regulate strawberry color. Strawberry color is mainly affected by anthocyanins [26]. In our study, TA treatment promoted the biosynthesis and degradation of chlorophyll, and the effect on synthesis may be greater than that on degradation. Because more genes in the carotenoid synthesis pathway, including the rate-limiting enzyme PSY, were down-regulated after TA treatment, we believe that TA treatment may inhibit carotenoid biosynthesis. Anthocyanins are ubiquitous in plants, a large number of colors result from the anthocyanin synthesis [50,51]. Anthocyanin biosynthesis is one of the branches of the flavonoid biosynthesis pathway [52,53]. In the anthocyanin biosynthetic pathway, phenylalanine ammonia-lyase (PAL) catalyzes phenylalanine to form cinnamic acid. Cinnamic acid is catalyzed to form coumaryl-CoA by cinnamic acid 4-hydroxylase (C4H) and 4-coumarate CoA ligase (4CL). Coumaryl-CoA and malonyl-CoA are catalyzed by chalcone synthase (CHS) to produce tetrahydroxychalcone. Tetrahydroxychalcone was catalysed by chalcone isomerase (chi), Flavanone 3-hydroxylase (F3H), flavanone 3’-hydroxylase (F3H) and flavanone 3’5’-hydroxylase (F3’5’h) to produce dihydroquercetin and dihydromyricetin. They are catalyzed by dihydroflavanone-4-reductase (DFR) to produce colorless anthocyanin. Colorless anthocyanin was synthesized by anthocyanidin synthase (ANS) [26,54,55]. ANS and UDP-glucose flavonoid 3-O-glucosyltransferase (UFGT) are responsible for the last step in the anthocyanin biosynthesis process. They work synergistically in the cytoplasm and vacuoles to convert unstable anthocyanins into stable anthocyanins [56]. In our study, the genes of key enzymes in anthocyanin biosynthesis, PAL, CHS, CHI, F3H, DFR, and ANS, were down-regulated and their activity decreased. UFGT down-regulated genes were more than up-regulated genes, and their activity was also decreased. Apparently, TA inhibited anthocyanin synthesis. We believe that TA retards the change of strawberry fruit color by promoting chlorophyll synthesis and inhibiting carotenoid and anthocyanin synthesis, thereby delaying fruit senescence.

Plant hormones regulate strawberry senescence. Ethylene is one of the most studied plant hormones that regulates mature aging. As a starter of mature aging, ethylene affects the transcription and translation of many related genes [7,57,58,59,60,61]. More and more studies show that ethylene plays an important role in regulating the maturity and senescence of non-jumping fruits [62]. Studies have shown that treating strawberries with 1-methylcyclopropene (1-MCP), an ethylene inhibitor, can reduce strawberry respiration rate, maintain the firmness and color of strawberry fruits after harvest.

Inhibiting the activity of phenylalnine ammonialyase (PAL) enzyme, increasing the activity of SOD and CAT, slowing the increase of anthocyanin and phenol content, and delaying the aging process [63,64,65,66]. This is very similar to our results. In our study, after TA treatment, the activity of ACO and ACS, the rate-limiting enzymes of ethylene biosynthesis, decreased, and the activity of most enzymes in ethylene signal transduction (ETR, SIMKK, EIN3, EBF1/2, and ERF1/2) were also reduced, and the results showed that TA treatment could inhibit ethylene biosynthesis and signal transduction, which may explain why TA delays the senescence of strawberry fruits. During the ripening and aging of strawberry fruits, the production of abscisic acid increased rapidly, and treatment with ABA increased fruit respiration and cellulase activity. ABA plays an important role in strawberry ripening and aging [67,68,69]. After TA treatment, the activities of key enzymes SDR and AAO3 in ABA biosynthesis increased. In ABA signal transduction, PYR/PYL and ABF were both down-regulated, and PP2C, a negative regulator of signal transduction, was up-regulated. Although TA promoted the synthesis of abscisic acid, it inhibited its signal transduction, which may be one of the important reasons for delaying strawberry senescence by TA treatment. Auxin can promote the early expansion of strawberry fruits. Auxin may delay the fruit maturation by activating the auxin signal and down-regulating the expression of cell wall degradation, anthocyanin synthesis and sucrose synthesis [70,71,72]. After TA treatment, during auxin signal transduction, most positive genes including auxin-responsive protein IAA (AUX/IAA) were up-regulated, and transport inhibitor response 1 (TIR1) was down-regulated. This indicates that TA treatment promoted IAA synthesis and auxin signal transduction, which may explain why TA retards strawberry senescence. In addition, GA, JA, SA, BR, and CTK signal transduction were also affected by TA.

As an important carbohydrate storage during fruit development, starch is very important in maintaining the fruit’s firmness and normal growth and development [31]. The activities of key enzymes SSY, GBSS, SS, GBE1 in starch synthesis were enhanced, and the enzyme BAM activity of starch hydrolysis was also enhanced. TA treatment promoted starch synthesis and degradation, which may have a greater effect on synthesis than degradation. The activities of EGs and bgl, which catalyzed cellulose hydrolysis, were reduced, and TA also inhibited cellulose degradation. The activity of SS was enhanced. It can catalyze both sucrose synthesis and sucrose decomposition. It is generally believed that the main function of SS is to provide precursors for polysaccharide synthesis in the direction of sucrose degradation. Sucrose synthase plays an important role in plant starch synthesis, improving plant stress resistance, and affecting plant growth [28,29,30]. In total, TA treatment not only affects sugar metabolism in fruits, but also has a positive effect in delaying softening and enhancing stress resistance.

## 4. Materials and Methods

### 4.1. Plant Material

Strawberry (*Fragaria* × *ananassa* ‘Sweet Charlie’) fruits of uniform size were used for TA treatment. Before flowering, the TA (50 μM) was sprayed on the strawberry plant, the plant was cared for and recorded every day for the flowering time, bud appearance, and the inflorescence length. After fruits started to show, they were marked with a brand to record the fruit development. When the fruit ripened, the physiological and molecular changes were measured. The achenes on the fruit surface were removed, and the leftover pulp was cut into 0.5–0.8 cm^3^ cubes frozen in liquid nitrogen and stored quickly at −80 °C until use.

IR fruit (16 days after flowering) was used for TA (50 μM) treatment to measure the effect of TA on fruit senescence. The TA fruit was sprayed on the surface of IR fruit and covered with silver paper, the control fruits were treated with an equal amount of ddH_2_O, then put them into the greenhouse, and perform fruit treatment every other day for a total of 3 times. After 16 days, the fruits were collected, the seeds were removed, the left pulp was frozen in liquid nitrogen and stored at −80 °C.

### 4.2. Determination of Soluble Sugars, Amino Acids, PA, Anthocyanins by HPLC

20 g sample was taken from storage at −80 ° C and ground into powder with liquid nitrogen. 10 mL of 80% ethanol and 0.5 g of powder were mixed, incubated at 80 °C with a water bath for 3 min, and centrifuged at 10,000 *g* for 10 min. The supernatant was collected into a 200 mL Erlenmeyer flask. The residue was washed with 10 mL of 80% ethanol twice with the above process, and then the supernatants were mixed. The remaining residue was filtered with 1 mL of 80% ethanol again and the filtrate was transferred to a new tube, and 5% α-naphthol of two drops was added into the filtrate. If there was no purple ring, suggesting that the sugar had been isolated from the sample completely. The mixed supernatant above was evaporated using boiling water, and the sugar was washed twice with distilled water of 20 mL, and the volume was 50 mL in total by added the distilled water to. In total, 2 mL of it was used for LC-18 solid phase extraction. Of these 2 mL, 1 mL was extracted and discarded, and the other 1 mL was collected and passed through a 0.45 mm membrane to measure the sugar content.

After that, HPLC condition was as follows: Agilent Technologies 1200 series, 6.5 × 3300 mm Sugar-Pak TM-1 column (Waters); ChemStation version B.02.01-SR2; ultrapure water as mobile phase, The flow rate was 0.4 mL min-1; the column temperature was 60 °C; the temperature of the refractive index detector was 50 °C; and the injection volume was 20 μL. The standard samples used were D-(+) Glc, D-(-) Fru, Suc (Sigma-Aldrich). The whole process was repeated three times. The measurement of PA and anthocyanins were described as by Fuleki and Francis [73,74].

### 4.3. The ABA, IAA, and Ethylene Contents Measurement

The pulp of strawberry fruits (*n* = 3) was used to analyzed the ABA and IAA content. For ABA and IAA extractions, 1 g of strawberry pulp was ground into powder with liquid nitrogen and added with 80% methanol (*v/v*) to mix. Then these was centrifuged at 10,000 *g* for 20 min. To remove the polar compounds, the Sep-Pak C18 cartridge (Waters) was used to elute the supernatants. After that, the supernatants were assayed as described by Zhang et al. [75]. The strawberry fruits (*n* = 3) were put in 200 mL glass jars in greenhouse at 25 °C for 2 h, and 1 mL of gas from the glass was drawn out and injected into the gas chromatograph for ethylene assay as described by Sun et al. [76]. The experiment was performed with three replications.

### 4.4. Total RNA Extraction, cDNA Library Construction and Illumina Sequencing

1 g of fruits was taken from the −80 °C refrigerator and ground into powder with liquid nitrogen, the modified cetyltrimethylammonium bromide (CTAB) method was used to extract total RNA, and DNase (Takara, Beijing, China) was added to remove DNA interference. RNA concentration was analyzed with a NanoDrop 2000 spectrophotometer (Thermo Scientific, Wilmington, NC, USA) and the total RNA integrity was analyzed using 1.0% agarose gel electrophoresis. The library was constructed using an RNA library preparation kit (New England BioLbs, Ipswich, MA, USA) (NEB), and quality was checked by an Agilent 2100 Bioanalyzer and ABI StepOnePlus real-time PCR system, and qualified cDNA libraries were used for RNA sequences Hiseq (Personalbio, Nanjing, China) Next-Generation Sequencing (NGS) performed paired-end (PE) sequencing on all samples. There was an average of 6 GB of data per sample.

## 5. Conclusions

This study identified a number of genes that were differentially expressed between control and triacontanol treatments, and outlined the effects of exogenous triacontanol on strawberry fruit growth, development, and senescence. TA could promote strawberry flowering and fruit development. Transcriptome analysis results show that exogenous triacontanol inhibits active oxygen accumulation by increasing antioxidant enzyme activity, down-regulating enzyme genes related to fruit softening and coloring, and regulating IAA, ETH, and ABA biosynthesis and signal transduction to delay senescence and triacontanol, and also promotes the early growth and development of strawberry fruits and enhances stress resistance. This can provide a reference for the study of strawberry storage and freshness.

## Figures and Tables

**Table 1:** Differentially expressed genes in antioxidant enzymes.

Genes	No. of Up-Regulated	No. of Down-Regulated	Sum
SOD	5	0	5
CAT	1	0	1
POD	14	8	22
PPO	1	5	6

Note: SOD, superoxide dismutase; CAT, catalase isozyme 1; POD, peroxidase; PPO, polyphenol oxidase.

**Table 2:** Changes in cell wall softening-related enzyme genes under TA treatmen.

Gene	No. of Up-Regulated	No. of Down-Regulated	Sum
Cx	1	6	7
α-Af	0	3	3
PE	9	23	32
PG	10	5	15
PL	3	3	6
β-Gal	7	6	13
EXP	8	13	21
XET	16	3	19

Note: Cx, cellulase; α-Af, alpha-L-arabinofuranosidase; PE, pectinesterase; PG, polygalacturonase; PL, pectate lyase; β-Gal, beta-galactosidase; EXP, expansin; XET, xyloglucosyl transferase.

**Table 3:** Differential expression genes related to Porphyrin and chlorophyll metabolism under TA treatment.

Gene	No. of Up-Regulated	No. of Down-Regulated	Sum
HemL	0	1	1
HMBS	1	0	1
UROD	3	0	3
PPOX	2	0	2
CPOX	1	0	1
chlD	1	0	1
chlI	3	0	3
chlM	2	0	2
DVR	1	0	1
FECH	2	2	4
HMOX1	1	1	2
HY2	4	0	4
chlG	1	0	1
CAO	1	0	1
HCAR	1	0	1
NOL, NYC1	2	0	2
chlP	2	0	2

Note: HemL, glutamate-1-semialdehyde 2, 1-aminomutase; HMBS, hydroxymethylbilane synthase; UROD, uroporphyrinogen decarboxylase; PPOX, protoporphyrinogen/coproporphyrinogen III oxidase; CPOX, coproporphyrinogen III oxidase; chlD, magnesium chelatase subunit D; chlI, magnesium chelatase subunit I; chlM, magnesium-protoporphyrin O-methyltransferase; DVR, divinyl chlorophyllide a 8-vinyl-reductase; FECH, protoporphyrin/coproporphyrin ferrochelatase; HMOX1, heme oxygenase 1; HY2, phytochromobilin:ferredoxin oxidoreductase; chlG, chlorophyll/bacteriochlorophyll a synthase; CAO, chlorophyllide a oxygenase; HCAR, 7-hydroxymethyl chlorophyll a reductase; NOL, NYC1, chlorophyll(ide) b reductase; chlP, geranylgeranyl diphosphate/geranylgeranyl-bacteriochlorophyllide a reductase.

**Table 4:** Differential expression genes related to Carotenoid biosynthesis under TA treatment.

Gene	No. of Up-Regulated	No. of Down-Regulated	Sum
PSY	0	2	2
crtISO	2	0	2
CrtR-b	1	0	1
VDE	0	1	1
CCS1	0	1	1
NCED	0	7	7
AAO3	2	0	2
CYP707A	3	3	6
AOG	1	0	1

Note: PSY, phytoene synthase; crtISO, prolycopene isomerase; CrtR-b, beta-carotene 3-hydroxylase; VDE, violaxanthin de-epoxidase; CCS1, capsanthin/capsorubin synthase; NCED, 9-cis-epoxycarotenoid dioxygenase; AAO3, abscisic-aldehyde oxidase; CYP707A, (+)-abscisic acid 8’-hydroxylase; AOG, abscisate beta-glucosyltransferase.

**Table 5:** Differential expression genes related to anthocyanins metabolism under TA treatment.

Gene	No. of Up-Regulated	No. of Down-Regulated	Sum
PAL1	0	8	8
CCoAOMT	1	0	1
CYP73A	0	4	4
CHS	0	7	7
HCT	0	5	5
C3’H	0	1	1
CHI	0	3	3
DFR	0	4	4
F3H	0	3	3
ANS	0	3	3
LAR	0	4	4
UFGT	5	6	11

Note: PAL1, phenylalanine ammonia-lyase 1; CCoAOMT, caffeoyl-CoA O-methyltransferase; CYP73A, trans-cinnamate 4-monooxygenase; CHS, chalcone synthase; HCT, shikimate O-hydroxycinnamoyltransferase; C3’H, 5-O-(4-coumaroyl)-D-quinate 3’-monooxygenase; CHI, chalcone isomerase; DFR, bifunctional dihydroflavonol 4-reductase/flavanone 4-reductase; F3H, naringenin 3-dioxygenase; ANS, anthocyanidin synthase; LAR, leucoanthocyanidin reductase; ANR, anthocyanidin reductase; UFGT, UDP-glucose flavonoid 3-O-glucosyltransferase.

**Table 6:** Differentially expressed genes of eight plant hormone synthesis and signal transduction.

Pathway	Gene	No. of Up-Regulated	No. of Down-Regulated	Sum
IAA biosynthesis	TAR	3	3	6
Auxin signal transduction	AUX1	0	1	1
	TIR1	0	2	2
	AUX/IAA	9	4	13
	ARF	0	1	1
	GH3	6	3	9
	SAUR	8	5	13
CTK signal transduction	CRE1	0	2	2
	AHP	0	1	1
	B-ARR	3	1	4
	A-ARR	5	1	6
GA signal transduction	PIF4	0	2	2
ABA biosynthesis	SDR	3	0	3
	AAO3	2	0	2
ABA signal transduction	PYR/PYL	1	8	9
	PP2C	5	1	6
	SnRK2	3	2	5
	ABF	0	1	1
ETH biosynthesis	ACO	4	5	9
	ACS	0	4	4
ETH signal transduction	ETR	0	4	4
	SIMKK	0	2	2
	MPK6	2	0	2
	EIN2	2	0	2
	EIN3	0	3	3
	EBF1/2	0	1	1
	ERF1/2	0	6	6
BR signal transduction	BKI1	3	0	3
	BSK	4	3	7
	CYCD3	1	3	4
JA signal transduction	JAR1	1	0	1
	COI1	0	1	1
	JAZ	4	6	10
SA signal transduction	TGA	4	8	12

Note: TAR, tryptophan aminotransferase-related protein; AUX1, auxin influx carrier; AUX/IAA, auxin-responsive protein IAA; TIR1, transport inhibitor response 1; ARF, auxin response factor; GH3, auxin responsive GH3 gene family; SAUR, SAUR family protein; CRE1, histidine kinase(cytokinin receptor); AHP, histidine-containing phosphotransfer peotein; B-ARR, two-component response regulator ARR-B family; A-ARR, two-component response regulator ARR-A family; PIF4, phytochrome-interacting factor 4; SDR, short-chain type dehydrogenase/reductase; AAO3, abscisic-aldehyde oxidase; PYR/PYL, abscisic acid receptor PYR/PYL family; PP2C, protein phosphatase 2C; SnRK2, serine/threonine-protein kinase SRK2; ABF, ABA responsive element binding factor; ACO, 1-aminocyclopropane-1-carboxylic acid oxidase; ACS,1-aminocyclopropane-1-carboxylate synthase; ETR, ethylene receptor; SIMKK, mitogen-activated protein kinase kinase 4/5; MPK6, mitogen-activated protein kinase 6; EIN2, ethylene-insensitive protein 2; EIN3, ethylene-insensitive protein 3; EBF1/2, EIN3-binding F-box protein; ERF1/2, ethylene-responsive transcription factor 1; BKI1, BRI1 kinase inhibitor 1; BSK, BR-signaling kinase; CYCD3, cyclin D3; JAR1, jasmonic acid-amino synthetase; COI1, coronatine-insensitive protein 1; JAZ, jasmonate ZIM domain-containing protein; TGA, transcription factor TGA.

**Table 7:** Differential expression genes related to starch and sucrose metabolism.

Gene	No. of Up-Regulated	No. of Down-Regulated	Sum
HK	1	0	1
SS	8	0	8
SSY	2	0	2
GBSS	3	0	3
PYG	2	0	2
GBE1	5	0	5
TREH	1	0	1
INV	0	3	3
EGs	0	6	6
UGP2	0	1	1
glgC	0	1	1
bgl	4	9	13
GN1_2_3	3	1	4
GN5_6	1	0	1
SPS	1	2	3
TPS	1	5	6
TPP	6	3	9
BAM	6	1	7

Note: HK, hexokinase; SS, sucrose synthase; SSY, starch synthase; GBSS, granule-bound starch synthase; PYG, glycogen phosphorylase; GBE1, 1,4-alpha-glucan branching enzyme; TREH, alpha, alpha-trehalase; INV, beta-fructofuranosidase; EGs, endoglucanase; UGP2, UTP-glucose-1-phosphate uridylyltransferase; glgC, glucose-1-phosphate adenylyltransferase; bgl, beta-glucosidase; GN1_2_3, glucan endo-1,3-beta-glucosidase 1/2/3; GN5_6, glucan endo-1,3-beta-glucosidase 5/6; SPS, sucrose-phosphate synthase; TPS, trehalose 6-phosphate synthase/phosphatase; TPP, trehalose 6-phosphate phosphatase; BAM, beta-amylase.

## Supplementary Materials

The following are available online at https://www.mdpi.com/2223-7747/9/4/488/s1, Table S1: High-quality sequence reads, Table S2: The number of genes in different expression level, Table S3: The identified differentially expressed genes, Table S4: GO enrichment of DEGs, Table S5: KEGG enrichment pathways of DEGs, Table S6: TF. family. count.

## References

[1] Wang W., Wang S., Wang M. (2019). Status and extension experience of protected strawberry cultivation technology in daxing district. J. Veg..

[2] Li D.D. (2019). Molecular Mechanism and Key miRNA Regulatory Factors of Strawberry Fruit Maturation Regulated by ABA.

[3] Lu W.J. (2018). Mechanisms of ABA and Auxin Regulating Banana and Strawberry Fruit Maturity.

[4] Alexander L., Grierson D. (2002). Ethylene biosynthesis and action in tomato: A model for climacteric fruit ripening. J. Exp. Bot..

[5] Pan Y.G., Xie J.H. (2009). Modern Postharvest Physiology of Fruits and Vegetables.

[6] Chen K.S., Zhang S.L. Research and regulation of functional genes for fruit maturation and senescence. Proceedings of the 4th Youth Symposium of the Chinese Horticultural Society.

[7] Zuo J.H., Chen A.J., Sun A.D., Luo Y.B., Zhu B.Z. (2010). Research advances in factors related to ripening and senescence of tomato fruits. China Agric. Sci..

[8] Liu D.S., Zhang Q., Lu D.H. (2001). Research progress of triacontanol in China and its application prospects in agriculture. China Eng. Sci..

[9] Ries S.K., Wert V.F., Sweeley C.C., Leavitt R.A. (1977). Triacontanol: A new naturally occurring pant growth regulator. Science.

[10] Mao J.Y., Yan Z.L. (2004). Principles and Practical Techniques of Plant Growth Regulation.

[11] Li W.H., Zhang S.J., Hou L.X. (2007). Effect of plant growth regulators on reducing pods poorness in summer soybean. Chin. Agric. Sci. Bull..

[12] Wert R.V. (1977). Growth responses of rice seedlings to triacontanol in light and dark. Planta.

[13] Wu G.N., Tang R.S., Zhang J.Y. (1986). Increasing yield of triacontanol. Plant Physiol. Newsl..

[14] Kumaravelu G., Livingstone V.D., Ramanujam M.P. (2000). Triacontanol-induced changes in the growth, photosynthetic pigments, cell metabolites, flowering and yield of green gram. Biol. Plant..

[15] Lu X.M., Zhu S.D. (2005). Effects of several pesticides on growth and resistance physiology of early-maturing edamame seedlings under water stress. J. Soil Water Conserv..

[16] Perveen S., Iqbal M., Parveen A., Akram M.S., Shahbaz M., Akber S., Mehboob A. (2017). Exogenous triacontanol-mediated increase in phenolics, proline, activity of nitrate reductase, and shoot k+ confers salt tolerance in maize (*Zea mays* L.). Braz. J. Bot..

[17] Yao W.C., Zhang C.Y., Liu A.R., Xu L. (2002). Effects of spraying chemical substances on wheat seedling growth and yield under drought stress. J. Anhui Agric. Sci..

[18] Wang A.L., Tang H.L., Wang Y.Y. (1995). Physiological effects of triacontanol (TA) on drought resistance and heat tolerance of wetland pine seedlings. Commun. Plant Physiol..

[19] Fan X.Z. (2004). Study on Physiological Mechanism of Triacontanol Influencing Rice Yield.

[20] Jin Y.M., Ma Y.F., You S.P., Lu D.Z. (1984). Physiological effects of triacontanol on senescence of in vitro leaves of hybrid rice. Commun. Plant Physiol..

[21] Wen Y.T. (2014). Study on the Fresh-Keeping Effect and~Mechanism of Different Plant Growth Regulators on Tulip Cut Flowers.

[22] Fan X.C., Guan J.F., Zhang J.Y., Liu C., Li G. (2003). Ca~(+)-ATPase activity and membrane lipid peroxidation level of microsomal membrane after strawberry harvest. Acta Hortic..

[23] Chen X.H., Zheng Y.H., Yang Z.F., Ma S.J., Feng L., Wang X.X. (2005). Effects of high oxygen treatment on active oxygen metabolism and decay of strawberry fruits after harvest. J. Nanjing Agric. Univ..

[24] Brummell D.A. (2006). Cell wall disassembly in ripening fruit. Funct. Plant Biol..

[25] Tao J., Zhang S.L., Zhang L.C., An X.M., Liu C.R. (2003). Relationship between the formation of citrus peel color and changes in carotenoid components. J. Plant Physiol. Mol. Biol..

[26] Jaakola L. (2013). New insights into the regulation of anthocyanin biosynthesis in fruits. Trendsin Plant Sci..

[27] Tang T.T., Xie X.F., Ren X., Zang J., Wang Z.D. (2020). Research advances in strawberry storage and preservation technology. Food Eng. Technol..

[28] Fang J.G., Zhu X.D., Jia H.F., Wang C. (2017). Research advances in physiological functions of plant sucrose synthase. J. Nanjing Agric. Univ..

[29] Dou Y. Biological function analysis of sucrose synthase SH1 in multi-enzyme complexes in regulating starch biosynthesis. Proceedings of the Cell Activity, Life Vitality—Summary of Abstracts of the Congress of All Members of the Chinese Society of Cell Biology and the Twelfth Academic Conference, Chinese Society for Cell Biology.

[30] Li L.N., Kong J.Q. (2015). Structure, function and application of plant sucrose synthase. Chin. J. Biochem. Mol. Biol..

[31] Zhou G.Z., Diao T.Q. (1997). Relationship between changes in starch content and amylase activity and storability of kiwi fruit. Fruit Sci..

[32] Lu D.Z., Fu J.R., Song S.Q. (1997). Plant Senescence and Its Regulation.

[33] Sun C.P., Zhang J.Z. (1999). Introduction to Free Radical Biology.

[34] Li X., Yue H., Wang S., Huang L.Q., Ma J., Guo L.P. (2013). Factors affecting plant antioxidant enzyme activity and its research hotspots and current status. China J. Chin. Mater. Med..

[35] He L.H. (2001). Polyphenol oxidase in higher plants. Plant Physiol. Newsl..

[36] Hu C.H. (2009). Research status of polyphenol oxidase. China High Tech. Enterp..

[37] Zhao F.X., Li Y.L., Hao G.P. (2019). Cloning and expression analysis of Cu/Zn SOD Gene in *Salvia miltiorrhiza* Bunge. Tradit. Chin. Med..

[38] Rao L.S., Xu S.S., Huang T.S., Huang L.Q., Ma J., Guo L.P. (2018). Analysis of Cu/Zn-SOD gene expression in Chinese fir under different stress. J. For. Environ..

[39] Ping L. (2016). Expression of Alfalfa Cu/Zn-SOD Gene in Petunia and Analysis of Its Cold Tolerance.

[40] Liao X. (2019). Tolerance Analysis of Rice Over-Expressing Superoxide Dismutase Gene OsCu/Zn-SOD Under Saline-Alkali Stress.

[41] Han S., Liu Y.F., Zhu T.H., Liu Y.G., Qiao T.M., Li S.J., Wang Y.L., Xu Y.L., Mo Y.Y. (2019). Cloning and prokaryotic expression of chestnut iron superoxide dismutase gene (CmFeSOD). Northwest Agric. J..

[42] Wei N. (2008). Effect of Pre-Warming Treatment on Cell Wall Degradation of Postharvest Grass.

[43] Abeles F.B., Takeda F. (1990). Cellulase activity and ethylene in ripening strawberry and apple fruits. Sci. Hortic..

[44] Guo M.L., Xue Y.C. (2016). Changes of several enzymes during strawberry senescence. J. Dalian Univ. Technol..

[45] Xie X.Z. (2018). Cloning and Expression of Arabinofuranosidase and Its Application in Wort Production.

[46] Suo B. (2006). Study on Degradation Characteristics of Cell Wall Polysaccharides During Softening of Peach Fruits.

[47] Yamaki S., Machida Y., Kakiuchi N. (1979). Changes in cell wall polysaccharides and monosaccharides during development and ripening of Japanese pear fruit. Plant Cand Cell Physiol..

[48] Iwai H., Ishii T., Satoh S. (2001). Absence of arabinan in the side chains of the pectic polysaccharides strongly associated with cell walls of *Nicotiana plum baginifolia* non-organogenic callus with loosely attached constituent cells. Planta.

[49] Zhang Y.W., Xin Y., Chen F.S. (2019). Research progress of pectin degrading enzymes and related genes during fruit softening. Preserv. Process..

[50] Koes R., Verweij W., Quattrocchio F. (2005). Flavonoids: A colorful model for the regulation and evolution of biochemical pathways. Trends Plant Sci..

[51] Zhai R., Wang Z.M., Zhang S.W., Meng G., Song L.Y., Wang Z.G., Li P.M., Ma F.W., Xu L.F. (2016). Two MYB transcription factors regulate flavonoid biosynthesis in pear fruit (*Pyrus bretschneideri* Rehd.). J. Exp. Bot..

[52] Holton T.A., Cornish E.C. (1995). Genetics and biochemistry of anthocyanin biosynthesis. Plant Cell.

[53] Petroni K., Tonelli C. (2011). Recent advances on the regulation of anthocyanin synthesis in reproductive organ. Plant Sci..

[54] Wu M., Liu J.L., Song L.Y., Li X.Y., Cong L., Yue R.R., Yang C.Q., Liu Z., Xu L.F., Wang Z.G. (2019). Differences among the anthocyanin accumulation patterns and related gene expression levels in red pears. Plants.

[55] Dubos C., Le Gourrierec J., Baudry A., Huep G., Lanet E., Debeaujon I., Routaboul J.-M., Alboresi A., Weisshaar B., Lepiniec L. (2008). MYBL2 is a new regulator of flavonoid biosynthesis in *Arabidopsis thaliana*. Plant J..

[56] Hou F.Y., Wang Q.M., Li A.X., Zhang H.Y., Dong S.X., Xie B.T. (2009). Study progress on anthocyanin synthetase in plants. China Agric. Sci. Technol..

[57] Zhang F., Ji S.J., Wei B.D., Cheng S.C., Zhou X., Zhou Q. (2020). Research progress of plant hormones affecting postharvest senescence of blueberry fruits. Packag. Eng..

[58] Lu L.F., Lu H.P., Wu C.Q., Fang W.W., Yu C., Ye C.Z., Shi Y.B., Yu T., Zheng X.D. (2013). *Rhodosporidium paludigenum* induces resistance and defense-related responses against *Penicillium digitatum* in citrus fruit. Postharvest Biol. Technol..

[59] Qu W.Y., Liu Z.Z., Xie L.M., Ao H. (2017). Regulation of exogenous abscisic ACID and ethephon on important quality of blueberry. Jiangsu Agric. Sci..

[60] Li H.X. (2019). The Effect of Abscisic ACID on Ripening and Softening of Raspberry Fruit and Its Relationship with Ethylene.

[61] Jiang A.L., Meng X.J., Hu W.Z., Wang Y.Y., Jiang B. (2011). Effects of exogenous ethylene treatment on sensory properties and respiratory metabolism of postharvest blueberry. Food Ind. Sci. Technol..

[62] Cheng R., Sheng J.P. (2015). Research advances on influencing factors of ripening and senescence of strawberry fruits and their regulatory mechanisms. Food Sci..

[63] Li Z.Q., Wang L.J., Gong W.H., Wang Z.H. (2006). Effects of 1-MCP on postharvest physiology and quality of strawberry fruits. J. Fruit Sci..

[64] Jiang Y.M. (2001). 1-Methylcyclopropene treatment affects strawberry fruit decay. Postharvest Biol. Technol..

[65] Bower J.H., Biasi W.V., Mitcham E.J. (2003). Effects of ethylene and 1-MCP on the quality and storage life of strawberries. Postharvest Biol. Technol..

[66] Shi L., Sheng J.P., Yu M.M., Ouyang L.Z., Sheng L. (2007). Effect of 1-MCP treatment on shelf-life quality of strawberries after storage. Sci. Technol. Food Ind..

[67] Wu Y.M., Gu C.Q., Tong G.F., Liu Y. (1992). The role of ABA and ethylene in ripening and aging of strawberry after harvest. Chin. J. Plant Physiol..

[68] Gu C.Q., Zhang B.C. (1992). Effects of ABA and ethephon on post-ripening and senescence of strawberry. J. Southwest Agric. Univ..

[69] Jia H.F., Chai Y.M., Li C.L., Dong Q.H., Qin L., Shen Y.Y. (2011). Analysis of FaABAR/CHLH expression changes and influencing factors of abscisic ACID receptor gene in strawberry fruit. Acta Hortic..

[70] Symons G.M., Chua Y.J., Ross J.J., Quittenden L.J., Davies N.W., Reid J.B. (2012). Hormonal changes during non-climacteric ripening in strawberry. J. Exp. Bot..

[71] Kang C.Y., Darwish O., Geretz A., Shahan R., Alkharouf N., Liu Z. (2013). Genome scale tran scriptomic insights into early-stage fruit development in woodland strawberry *Fragaria vesca*. Plant Cell.

[72] Chen J.X., Mao L.C., Lu W.J., Ying T.J., Luo Z.S. (2016). Transcriptome profiling of postharvest strawberry fruit in response to exogenous auxin and abscisic ACID. Planta.

[73] Fuleki T., Francis F.J. (1968). Quantitative methods for anthocyanins. 1. Extraction and determination of total anthocyanin in cranberries. Food Sci..

[74] Fuleki T., Francis F.J. (1968). Quantitative methods for anthocyanins. 2. Determination of total anthocyanin and degradation index for cranberry juice. Food Sci..

[75] Zhang M., Yuan B., Leng P. (2009). The role of ABA in triggering ethylene biosynthesis and ripening of tomato fruit. Exp. Bot..

[76] Sun J.H., Luo J.J., Tian L., Li C.L., Xing Y., Shen Y.Y. (2013). New evidence for the role of ethylene in strawberry fruit ripening. Plant Growth Regul..

